# Field and Laboratory Studies Provide Insights into the Meaning of Day-Time Activity in a Subterranean Rodent (*Ctenomys* aff. *knighti*), the Tuco-Tuco

**DOI:** 10.1371/journal.pone.0037918

**Published:** 2012-05-23

**Authors:** Barbara M. Tomotani, Danilo E. F. L. Flores, Patrícia Tachinardi, José D. Paliza, Gisele A. Oda, Verônica S. Valentinuzzi

**Affiliations:** 1 Departamento de Fisiologia, Instituto de Biociências, Universidade de São Paulo, São Paulo, São Paulo, Brazil; 2 Centro Regional de Investigaciones Científicas y Transferencia Tecnológica (CRILAR), Anillaco, La Rioja, Argentina; Simon Fraser University, Canada

## Abstract

South American subterranean rodents (*Ctenomys* aff. *knighti*), commonly known as tuco-tucos, display nocturnal, wheel-running behavior under light-dark (LD) conditions, and free-running periods >24 h in constant darkness (DD). However, several reports in the field suggested that a substantial amount of activity occurs during daylight hours, leading us to question whether circadian entrainment in the laboratory accurately reflects behavior in natural conditions. We compared circadian patterns of locomotor activity in DD of animals previously entrained to full laboratory LD cycles (LD12∶12) with those of animals that were trapped directly from the field. In both cases, activity onsets in DD immediately reflected the previous dark onset or sundown. Furthermore, freerunning periods upon release into DD were close to 24 h indicating aftereffects of prior entrainment, similarly in both conditions. No difference was detected in the phase of activity measured with and without access to a running wheel. However, when individuals were observed continuously during daylight hours in a semi-natural enclosure, they emerged above-ground on a daily basis. These day-time activities consisted of foraging and burrow maintenance, suggesting that the designation of this species as nocturnal might be inaccurate in the field. Our study of a solitary subterranean species suggests that the circadian clock is entrained similarly under field and laboratory conditions and that day-time activity expressed only in the field is required for foraging and may not be time-dictated by the circadian pacemaker.

## Introduction

Daily photic entrainment mechanisms of circadian systems have been traditionally studied in the laboratory settings, where light-dark cycles are artificially controlled. However, the large discrepancy between laboratory and natural lighting conditions and the species-specificity in daily light exposure patterns has also motivated studies that check whether entrainment mechanisms are similar under those different conditions. This concern is particularly important when studying animals that live in dens or burrows, including many rodent species. DeCoursey [1] measured the activity of flying squirrels (*Glaucomys volans*) in simulated den systems to achieve a model of natural entrainment based on daily “light-sampling” patterns in the laboratory setting. Hut et al. [2] addressed this same question outside the laboratory by measuring the activity of ground squirrels (*Spermophilus citellus*) that were released into semi-natural enclosures carrying light sensors. These studies provided new perspectives on entrainment research and showed that animals with unusual light exposure patterns can be useful models for understanding synchronization in nature.

The underground environment offers unique opportunities for circadian research, due to its presumed constant darkness and low amplitude of other environmental cycles. Subterranean animals are interesting candidates for this line of research because even synchronization to day-night and the occurrence of light exposure is uncertain in nature.The circadian organization of subterranean species may provide interesting insights into whether and how synchronization to the external day and night occurs in this poorly cyclical natural environment [3] [4]. Members of the South American genus *Ctenomys*, popularly known as “tuco-tucos”, comprise the greatest number of species among subterranean rodents with more than 60 species ranging from 12° south latitude to Patagonia [5], [6]. Previous laboratory studies with solitary tuco-tucos from La Rioja, Argentina (*Ctenomys* aff. *knighti*) have shown that this species are clearly nocturnal under conditions of LD 12∶12 (12 hours of light and 12 hours of dark), constant temperature and *ad libitum* food [7]. Notably, upon release of these animals into DD conditions, a rhythm with 24 h period persists for several days before attaining its free-running value, which is greater than 24 h. These “aftereffects” of laboratory entrainment [8]–[11] are also noticeable in bats as a result of natural entrainment [12]. We conducted a comparative study of rhythmicity in DD, of animals previously entrained to full laboratory LD cycles (LD12∶12) against animals that were trapped directly from the field. In both cases, the initial phase and the long-lasting 24 h period of activity rhythms indicated that animals had been previously synchronized, displaying aftereffects of laboratory and field entrainment, respectively. This comparison suggested that the circadian clock is entrained similarly under field and laboratory conditions.

To verify the temporal light exposure patterns that allowed entrainment of subterranean rodents in the field, individuals were continuously observed in semi-natural enclosures during day-light hours. These observations revealed that tuco-tucos express considerable amount of aboveground activity during day-light hours, which is a time with no counterpart in the laboratory conditions. Moreover, these activities comprise foraging and soil removal behaviors, which are typically from the field. By combining the data gathered observing a South American wild species both in the field and in the laboratory, we provide novel elements to the recently generated views regarding the meaning of diurnal/nocturnal divisions displayed by rodents in the field [13]–[19].

## Materials and Methods

### a) Ethics Statement

The capture and laboratory experimentation protocols were approved and authorized by the Legal and Technical board (*Oficina de Técnica legal*) of the Environmental Department of La Rioja (*Secretaria de Ambiente, Ministerio de Producción y Desarrollo Local*), permission n° 062-08. Every procedure of this study followed the guidelines of the American Society of Mammalogists for animal care and handling [20].

### b) Study location

*Ctenomys* aff. *knighti* is found in the province of La Rioja, Argentina (26°48′S; 66°56′W; 1,445 m). This location's arid climate has a mean annual rainfall ranging from 100–200 mm that is almost exclusively limited to the summer months (between December and February) [21]. The soil is sandy and poor, and the predominant vegetation is a shrubby steppe with characteristic flora from the Zygophyllaceae, Fabaceae and Cactaceae families [21], [22]. There are also extensive grape, walnut and olive plantations that *C.* aff *knighti* seems to occupy as successfully as natural areas; not only does the local community consider this species to be an agriculture plague, but the animals frequently leave fresh mounds and are easily captured in these cultivated areas.

### c) Animals

The animals found in the study area were first identified as *Ctenomys knighti* Thomas, 1919. A final identification is currently being confirmed with karyotypic and genetic analysis at the *Grupo de Investigaciones de la Biodiversidad (GIB) IADIZA-CCT Mendoza-CONICET*. Additionally, skins and skeleton samples of these animals were sent to three Argentinean Natural Science Museums: *Centro Nacional Patagónico-CENPAT, Puerto Madryn, Chubut* (specimens CNP-2429 to -2432), *Colección de Mamíferos de la Fundación Miguel Lillo, Tucumán* (still unnumbered) and *Colección Mastozoológica del IADIZA, Mendoza* (still unnumbered). The animals were live-trapped within a 15 km^2^ area surrounding the laboratory, with buried traps constructed from a 25-cm long PVC plumbing pipe with a 7.5-cm outer diameter. The traps were set by opening a burrow beneath a fresh mound of soil and positioning the pipe horizontally along the floor of the tunnel. Because the animals sometimes plugged the traps with loose soil, the traps were checked every 1–2 h, cleaned and reset as needed.

### d) Laboratory constant conditions

To facilitate animal care, the laboratory was maintained in “constant darkness” that actually consisted of a dim red light with an intensity of 1–5 lux, which corresponds to the full moon at night. This illumination was provided by two incandescent red lamps (Philips 40/25 W) connected to a dimmer (200 W, Teclastar Milano, San Martín, Buenos Aires, Argentina). The temperature was maintained at 23±2°C. Food (carrots, sweet potatoes, lettuce, rabbit pellets, sunflower seeds and/or grass) was offered every day at random times. Tuco-tucos obtain water exclusively from food; therefore, it was not necessary to provide water [23]. The animals were housed individually either in acrylic cages (53×29×27 cm) with computer monitored running wheels (23 cm in diameter, 10 cm wide, 1 cm between the bars) or in glass cages (37×26×21 cm) with infrared motion sensors located in the middle of the cage lid. The cages were filled with a layer of shredded paper and cleaned weekly at random times.

General motor activity detected by the infrared sensors and wheel-running activity were both continuously recorded with the ArChron Data Acquisition System (Simonetta System, *Universidad Nacional de Quilmes, Buenos Aires*) at 5-minute intervals. Graphical output (actograms) and rhythm analysis were carried out using the *El Temps* software (A. Díez-Noguera, *Universitat de Barcelona*, 1999). The mean activity onset was calculated by fitting a line through 3–5 onsets before the rhythm began to free-run. Student's t-test was used to compare the average onset of each group (wheel-running activity and infrared sensors).

### e) Semi-natural enclosure

An outdoor enclosure was built in a rural area that is naturally occupied by wild tuco-tucos. The enclosure measured 10 m×5 m and was protected with wire mesh on top and sides (1.2 m above-ground and 1 m underground) to keep foxes and flying predators away. The enclosure was designed to accommodate only one animal at a time because this species is strictly solitary. Using an enclosure is the best and most controlled way to follow the behavior of a single animal because tuco-tucos are small, have a sandy color that easily blends with the environment and often emerge unpredictably from new holes. The size of the enclosure was based both on the home-range size determined for *C. talarum* [24] and our telemetry-based area estimation of *C.* aff. *knighti* during the summer. Upon release, each animal readily excavated its own burrow systems.

A meteorological station located only 80 m away from the field enclosure allowed the recording of the ambient temperature, wind speed, rain and humidity during observation days. Exclusively during the 2011 summer observation phase, the environmental temperature was continuously measured at a fixed 60-cm underground location inside the burrow using HOBO data loggers U10/003 (Onset Computer Corporation, Bourne, MA).

### Experiments

#### Experiment 1

Tuco-tucos (n = 10, 7 adult males and 3 females) were trapped directly from the field and were immediately placed in the laboratory under constant conditions. The trapping was conducted from late May to August 2010 at randomly distributed times throughout the day. By allowing half of the animals access to a running wheel, it was also possible to verify any effect of wheel running in phase determination.

Additionally, a second group of five adult laboratory animals (2 males and 3 females) kept in wheel-running cages was synchronized to a standard full 12∶12 h LD cycle and then transferred to the same constant dim light conditions as the field-captured animals. This allowed us to compare synchronization in the field with synchronization in the laboratory.

#### Experiment 2

Three adult, non-pregnant females were individually observed during March 2010, July 2010 and March 2011. A continuous observation was maintained during daylight hours (from 06:30 to 20:30 in March (summer) and from 07:50 to 19:10 in July (winter)), and both the timing of surface emergence and a behavioral description were recorded with 1 minute precision. This implied 400 hours spent by 6 persons working in 3 hour shifts, in continuous, highly alert observation.

These observations started 12, 4 and 6 days, respectively, after the release of the animals into the enclosure. Importantly, the environmental conditions differed among the 3 observations, with one dry and one rainy March month, in 2010 and 2011, respectively (Table 1). During the 2010 dry March observation, plants collected outside the enclosure were delivered daily on the surface. No external food was offered in the March 2011 humid summer observation. As for the July observation, sunflower seeds were offered only during the first 4 days. Very few of the offered items were, apparently, transported into the burrows.

**Table 1:** Environmental conditions and food availability levels of the three observation seasons.

	food availability	mean relative humidity (%)	total rain (mm)	temperature (°C)			wind speed (km/h)		
				mean (±SD)	max	min	mean (±SD)	max	min
Summer 2010 (March 01–March 11)	scarce	32.3	0	22.7 (±4.12)	31.6	15.4	6.7 (±3.13)	17.7	0
Winter 2010 (July 26–August 06)	scarce	14.6	0.01297 (rain and snow)	5.66 (±5.21)	18.6	−2.1	4.13 (±2.85)	12.9	0
Summer 2011 (March 01–March 10)	abundant	32.3	11.5	20.73 (±3.21)	28.5	15.1	4.52 (±2.2)	11.3	0

At the end of each series of observations, animals were transferred directly from the field enclosure to constant laboratory conditions and their activity was monitored by infrared motion sensors. The lab is located within walking distance from the enclosures. The March 2010 animal was kept in this constant dim red light for 15 days, the July 2010 animal for 10 days and the March 2011 March animal for 5 days.

## Results

### Experiment 1

**Figure 1:**
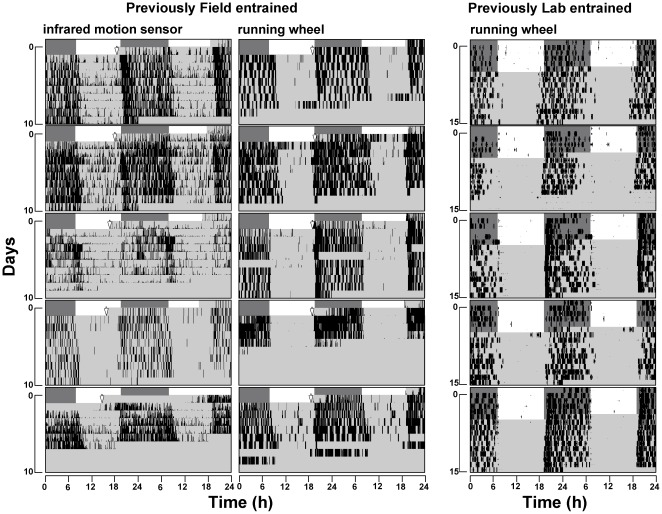
Comparison of activity phase in constant lab conditions of animals entrained previously to lab and field conditions. Double-plotted actograms show infrared motion detected general activity of five individuals (left panel), and wheel-running activity of another five individuals (middle panel) that were trapped directly from the field. Laboratory animals (right panel) show wheel-running activity entrained to LD 12∶12 and then released into constant conditions. In all cases, activity was concentrated in the phase corresponding to night in the field or dark phase in the lab (from 19:00 to 07:00). Vertical arrows indicate the time when the animal was released into its lab cage and activity measurement began. The light gray background represents the constant darkness of the lab, while the dark gray and white backgrounds represent the timing of natural light/dark cycles (civil twilights according to the U.S. Naval Oceanography Portal, www.usno.navy.mil).

Fig. 1 (left and middle panel) shows the double-plotted actograms of animals trapped in the field and brought directly to the constant laboratory conditions. All animals displayed 24 h rhythms under constant conditions indicating that they had been entrained in the field. Regardless of the presence of running wheels, the 24 h activity of all animals was concentrated in the phase corresponding to night in the field. Moreover, when a line was fitted through the onsets and offsets, the average onset of animals with activity that was measured by infrared motion sensors was 19 h 30 (±16 min), and the average offset was 8 h 43 (±24 min), while animals with access to running wheels displayed an average onset of 19 h 30 (±17 min) and an average offset of 8 h 48 (±51 min); there was no significant difference between the two conditions (Student's t-test: onset p = 1, offset p = 0,8), discarding possible influence of wheel access [19] in the phase of activity, in our species.

The right panel of Fig. 1 shows the five control animals that were previously entrained to a full LD cycle in the lab (showing clear synchronized nocturnal wheel-running activity), and when placed in DD maintained the 24 h period and the same night concentrated activity for the following 10 days.

This comparison of activity phase of previously field and laboratory entrained animals, indicates that synchronization is similar in both conditions. That is, tuco-tucos exhibited robust activity during the night, or dark phase of the previous entrainment cycle in both conditions, and activity onsets in DD corresponded to the beginning of dark on the entraining cycles, without transient changes.

### Experiment 2

**Figure 2:**
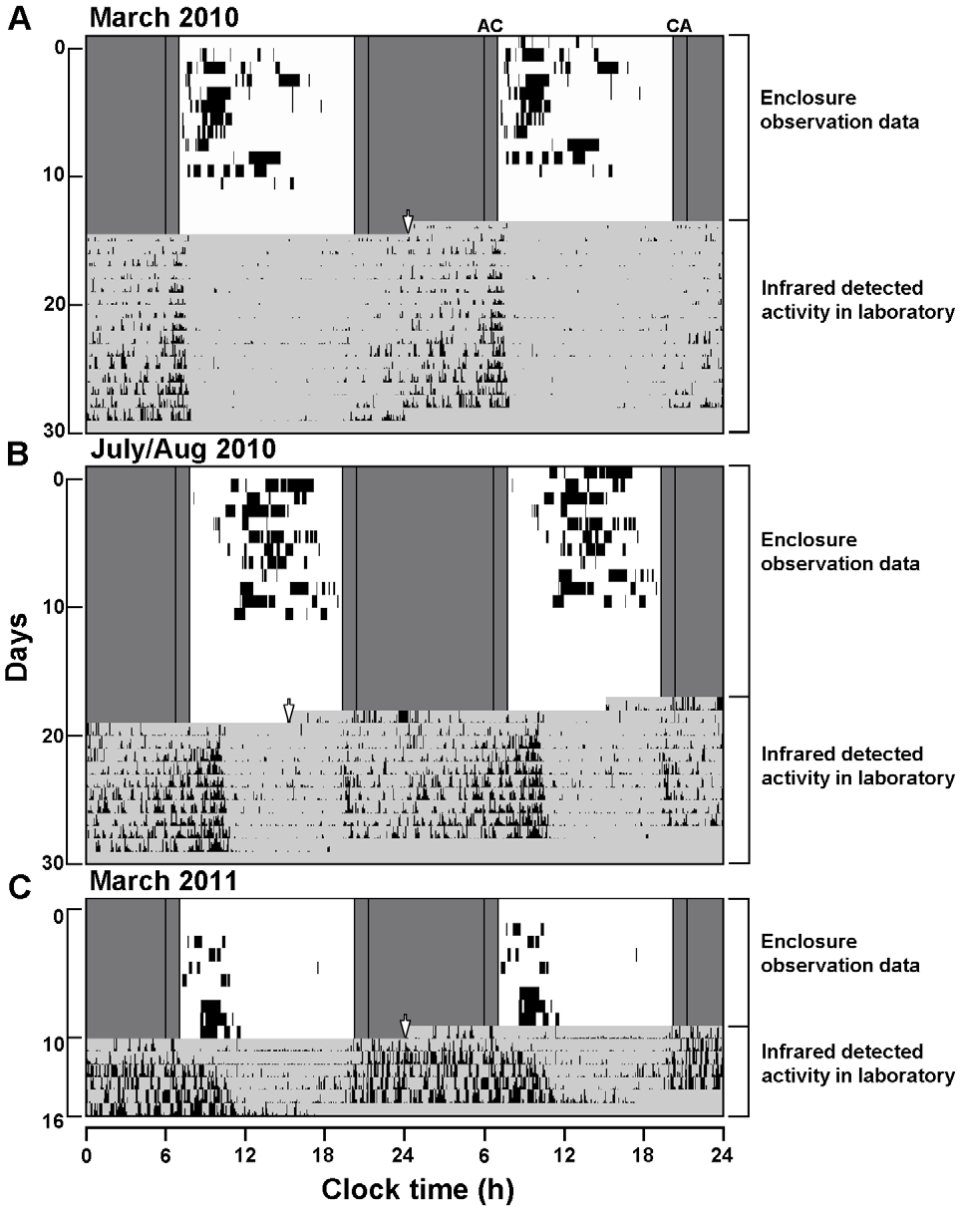
Phase of activity of the three enclosure entrained animals transferred from the semi-natural enclosure to constant lab conditions. A) March 2010 animal; B) July 2010 animal; C) March 2011 animal. Each actogram consists of two sections: upper section shows aboveground emergence times (black marks) of one individual animal during enclosure observation; lower section shows subsequent infrared-detected activity under constant laboratory conditions. Vertical arrows indicate the moment when the animal was released into its lab cage and infrared-based motion detection was initiated. In the upper sections of each figure, the dark gray and white backgrounds represent the timing of natural light/dark cycles. In the lower sections, the light gray background represents constant darkness (dim red light). Vertical lines show the astronomical (A) and civil (C) twilights according to the U.S. Naval Oceanography Portal (www.usno.navy.mil). Differences in the interval between the last observed activity in the enclosure and the first detected activity in the laboratory are caused by the differences in the time needed to trap each animal.

In all our 3 continuous observations, subterranean tuco-tucos emerged above-ground very often, exposing themselves to light on a daily basis. The upper actogram sections of Fig. 2 show the daily light exposure patterns during 10 days in each of the three observations. This light exposure occurs in short, randomly distributed episodes that may last from a few minutes to one hour.

The most perplexing feature of a total of 30 observation days was the high frequency of surface emergences that were observed during the day, which were not expected for a nocturnal, subterranean animal. Some freely living individuals were also seen sporadically (Table S1), as well as neighborhood vocalizations heard during day-light hours outside the enclosure every day. Our continuous observations revealed that tuco-tucos exposed themselves to light to accomplish two vital activities. First, the animals emerged above-ground to forage. In most of the episodes, they alternated between a vigilant posture and brief excursions toward plants located close to the opening of the burrow. During several excursions, they carried whole branches with leaves back to their burrows. While burrow openings were not sealed, animals were expected to re-emerge either a few minutes or several hours later. One or two openings were used each day, and some were reopened on subsequent days. New earth mounds early in the morning, indicative of night-time emergence, were only detected once in a total of 30 day observations. Second, the animals exposed themselves to light while vigorously throwing out earth in long soil removal episodes that could last more than 60 minutes. This behavior is presumably the final step of an underground behavior of excavation of new paths leading to new tunnel openings [25]. During the July 2010 observation, one freely living animal was observed to emerge in the outside vicinity of the enclosure, performing a similar 40-minute average daily soil removal episode during three consecutive days (Table S1). Other several excavation and foraging episodes occurred daily clearly indicating that the observed animals exhibited an important day-time activity component in the field.

**Figure 3:**
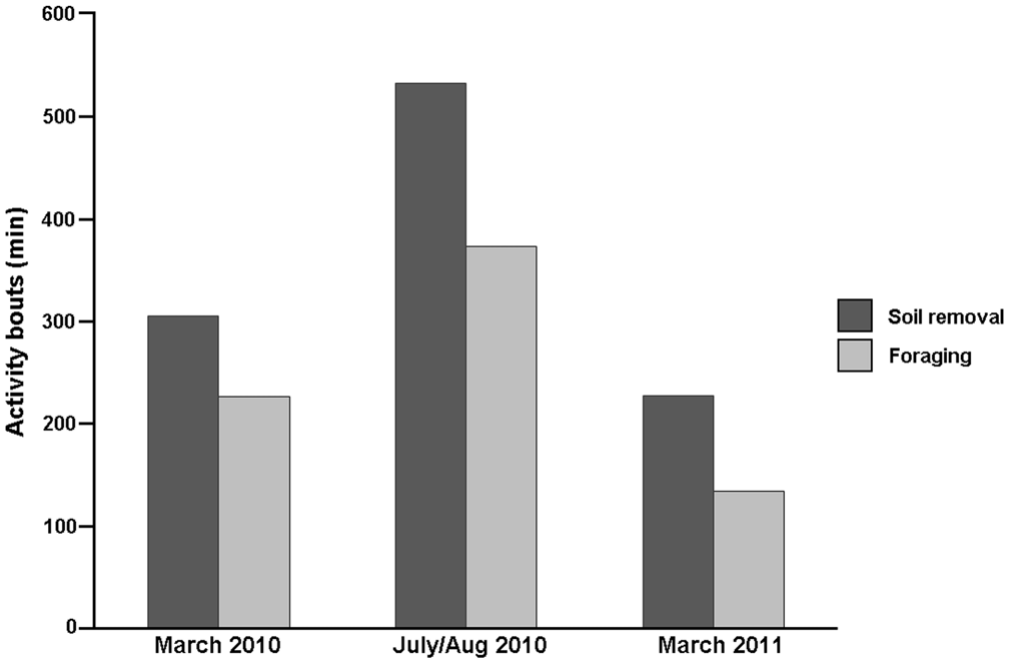
Total time spent in each above-ground activity component (soil removal or foraging) during the entire time of observations.

Our March, (summer) observations occurred exactly one year apart in radically different environmental conditions. Rains in the study area are variable among different years, but are always low (100–300 ml/year), and the climate, consequently, is usually dry. Nevertheless, the summer of 2011 was exceptionally rainy; consequently, the amount of vegetation in the study area was spectacularly much higher than in the previous year. Under this new condition, tuco-tucos exhibited fewer excursions, carrying a greater amount of plant material to the burrows. As a consequence, less time was spent in foraging, compared to the other observations (Fig. 3). In every observation, animals spent more time removing soil from their burrows than foraging.

Although the direct modulation of above-ground emergence by environmental factors was not investigated systematically, observation during March 2011 (summer) suggested that the peak of above-ground emergence may be correlated in time with lower underground temperatures (Fig. S1). Furthermore, two occurrences are worth noting. During the third day of the March 2010 observation , the animal emerged during the afternoon, which was unusually late, compared to the other days for this individual (day 3 in Fig. S2). Coincidently, the wind was unusually high during that morning. This was witnessed by the observer, who described high levels of wind and harsh, dusty conditions at the site and was later confirmed with the regional wind parameters obtained by the meteorological station (Fig. S2). As for the influence of rain, no emergence at all was observed during one rainy day during March 2011. Interestingly, this event was followed by intensified soil removal activity on subsequent days (Fig. S3). Rains are known to fill pores in the soil, decreasing the gas exchange capacity and consequently favoring a decrease in oxygen content and increase in carbon dioxide in inhabited tunnels. This has been suggested to induce digging activity in subterranean rodents to replenish burrow atmosphere [26].

Animal weights before release into the enclosure and at the end of each observation (March 2010, July 2010 and March 2011) were: 186.4 g/165.5 g, 141 g/128.8 g, 142 g/125.7 g, respectively.

Similarly to Experiment 1, upon transference to constant DD conditions after each of the 3 observations, animals displayed robust 24 h rhythms, indicating aftereffects of previous field entrainment (Fig. 2, lower actogram). The lack of enclosure data for several days before activity was monitored in the lab is due to the time it normally takes to capture an animal in the enclosure. It does not invalidate our conclusions, because the animals had been in the same naturally cycling environment. Activity was again concentrated in the phase corresponding to night in the field with no signs of transients. This result, as in Experiment 1, again indicated that the circadian oscillator had been entrained in the field-enclosure condition in the same way as the LD laboratory entrained animas and consequently the timing of observed intense field day-activity was probably not dictated by the circadian pacemaker.

## Discussion

Our observations revealed that subterranean tuco-tucos emerge to the surface frequently and expose themselves to light during these surface emergences that occur at randomly distributed times during the daylight hours. This is distinct from what is known for other subterranean species such as mole-rats which hardly expose themselves to the above-ground environment [25], [27]. Close examination of the behavioral repertoire of three tuco-tucos revealed that animals emerge during the day mostly for foraging and soil removal (burrow maintenance). On one hand, the timing of these behaviors is of interest. On the other hand, and most importantly, these behaviors are not allowed in the laboratory condition, being expected in nature but not in a cage. Because we cannot state that tuco-tucos are “diurnal” in the field or enclosures, we limit our discussion over the appearance of activity during the day-time. Surprisingly, when our day-active enclosure animals were transferred to constant laboratory conditions, their day-time component disappeared but rhythmicity persisted with activity concentrated in the hours corresponding to night in the field. The generality of this finding was shown by the other 10 animals that were trapped directly from the field and transferred to constant laboratory conditions. The night-time activity in DD displayed no transients, with a 24 h period that is notably different from their free-running period [7]. This period aftereffect is a hallmark of previous entrainment to an external cycle, which we previously observed in tuco-tucos recorded in LD and then in DD for many weeks [7]. It is a long-lasting modificationon the free-running period of the circadian oscillator and decays slowly in constant conditions [8]–[11]. In other words, it can be measured in the present time, under constant conditions, to assess if the animal was previously entrained. Most importantly, because the activity phase of this 24 h period rhythm is restricted to external night hours, both in previously lab and field entrained groups, it is concluded that the circadian oscillator is equally entrained in these two conditions (Fig. 1) and that day-time activity in the field contradicts the expected diurnal inactivity that is supposedly signaled by the circadian pacemaker [28].

There are at least 3 possible mechanisms responsible for timing of daytime activity observed in the field. One possibility is that these specific behaviors are controlled by the SCN pacemaker, but are expressed at a different phase relative to other waking behaviors. This possibility could not be further evaluated in the laboratory because the cages used for recording activity did not provide the opportunity to observe foraging or soil removal behaviors.

A second possibility is that the daytime activities observed in the field were not clock-controlled but were due to masking [29]–[31]. In this case, an environmental factor in the field would enhance expression of activity during the day or presumably inhibit during the night, without affecting the phase of the circadian pacemaker [16], [32], [33]. For instance, the behaviors could be due to masking effect of light, although different from behavioral masking effects normally observed in nocturnal animals. In this case, light would be interpreted as selectively stimulating foraging activity, whereas in laboratory studies of nocturnal rodents, light typically inhibits general activity [31], [34]. This can be evaluated in future studies. It is also possible that daytime activity is favored by other environmental factors that differ considerably between lab and field conditions such as the extreme daily temperatures of the desert [35], [36] and nocturnal predators [37]. Hints of environmental factors with the ability to modulate activity expression in tuco-tucos are presented in our Supplementary Material. Our observation did not provide information about what is happening during the night and underground. At most, we know that trap occlusion and burrow entrance closures were significantly more frequent in the hours between 4 am and 4 pm than for the rest of the day, occurring mostly between 8 am and 4 pm (M. Ralph, personal observation). It is thus possible that clock-controlled general night activity is normally expressing in the field (mostly underground) while particular behaviors (foraging and digging) are more heavily influenced by masking than others, being enhanced also during the day-time. Conversely, we cannot discard masking in the laboratory context, where, trapped in a cage, the natural foraging and burrowing behaviors become impossible.

A third possible mechanism controlling daytime activity in the field and alternative to masking is suggested by recent laboratory studies employing a “work for food” model of foraging behavior [17]. This model has been supported by experiments with mice carrying general-motion sensors in semi-natural enclosures [18] and seems to fit our wild species data nicely. This new model is based on an experimental laboratory protocol that explores the effect of food restriction and the subsequent increase of energy expenditure for obtaining food. This was accomplished by increasing the workload required to find food, which surprisingly caused nocturnal mice under LD cycles to intensify their activity during increasing portions of daylight hours. This perplexing behavioral change was reversed almost instantly when animals were transferred to DD with food offered *ad libitum*. In this new condition, activity became restricted to the time corresponding to the previous dark phase. Interestingly, the gross component of activity remained nocturnal throughout most of the experiment. Diurnal bouts arise as distinct components that seemingly dissociate from the main nocturnal activity bout, shifting into the middle of the light phase as the workload for food increases.

In our study, food was offered *ad libitum* in laboratory conditions, whereas it was mostly collected naturally by the animal in the field-enclosure. In this sense, the “working for food” model is consistent with the main features of our data for several reasons. First, it is consistent with activity restricted to the dark phase in the lab LD 12∶12 and *ad libitum* food conditions [7] while expressing during the day in our field enclosure observations. Second, it is consistent with the immediate disappearance of day-time activity when field-entrained animals are transferred to the lab under DD and *ad libitum* food conditions. Finally, and, most importantly, the day-time above-ground activity that consisted of foraging and soil removal could be strongly linked to food collection. Although tuco-tucos from the study area have been reported to also feed on underground roots [22], aerial plant parts such as leaves have been shown to be important food sources for other *Ctenomys* species [25].

Tuco-tucos adopt a foraging strategy of collecting only plants that are close to their burrow openings, and the animals are seemingly able to detect above-ground plants through olfaction [38]; thus, underground tunnel extensions are required when food is scarce [23]. Tunnel extensions imply more digging followed by more soil removal, thus increasing the energy expenditure [23], [39], [40]. Although our observations were limited to the daylight hours and we did not have access to the underground activity, an increase in above-ground soil removal activity, proportional to food scarcity was indicated in Fig. 3. When food is abundant, the observed tuco-tuco even reuse the same burrow openings in subsequent days, which requires less work. Thus, subterranean and herbivorous tuco-tucos are not only interesting models for light-entrainment studies, but they may also offer concrete ecological counterparts to the “working for food” paradigm [17].

In contrast to field masking, the model presented by Hut et al. [17] assumes that the diurnal component of activity is under the temporal control of another oscillator that is a “slave” with respect to the main pacemaker in the suprachiasmatic nuclei [41]. The “master-slave” relationship in circadian organization was proposed by Pittendrigh [42] to make sense of independent, adaptive phase adjustments of single physiological components within the circadian program. The results of Hut et al. [17] indicate that day- and night-active individuals within a species could arise due to a change in the phase relationship between the master circadian pacemaker and the slave oscillator elicited by different food availability levels in the environment. In this sense, the timing of rhythmic activity expression is not exclusively dictated by the circadian pacemaker, and this proposal has been indicated by several other works in distinct contexts [15], [43]–[46].

Induced sustained day-activity in nocturnal animals is not uncommon. It has been shown that rats trained daily during the light phase in a demanding cognitive task become clearly diurnal [47]. The main difference between this work and our data is the fact that daily task training can act as an entraining cycle. This was proved by activity starting at the previous light phase after training cessation and DD release, and subsequent resynchronization to the reinstated LD cycle, with clear transients from the diurnal component to nocturnal activity phasing. In our case, activity is concentrated in the previous dark phase soon upon release into DD, with notably no transients. Additionally, it is interesting to mention that in the data of Gritton et al. [47] the controls submitted to non-cognitive components of the same task did not show real entrainment but did show associated day-activity. In other words, our data could be more related to the control groups in which certain relevant external stimuli (water restriction, handling, etc) stimulate activity [47] in a phase that the circadian clock is dictating inactivity [28].Other explanations could also fit to our data and further studies are necessary in order to arrive at a firm model.

## Supporting Information

Figure S1 **Daily variation of environmental temperatures.** Mean values (denoted by squares) of registrations during the days indicated in Table 1: March 2010 (top), July 2010 (middle) and March 2011 (bottom). The underground temperature (60 cm deep) is included in March 2011 data (bottom) (small triangles). The timing and frequency of surface emergences of the tuco-tucos is indicated by vertical bars.
(TIF)

Figure S2 **Surface emergence timing (black bars) and wind speed (black line) data for the March 2010 observation.** The arrows indicate the moments where wind potentially acted as a masking agent for surface activity. Note: Data on days 4 and 5 was lost, due to equipment failure.
(TIF)

Figure S3 **Sum of the total time spent in distinct above-ground activities (soil removal and foraging) during the March 2011 observation.** The amount of rain is shown on top of the corresponding bars; no value indicates that no rain was registered.
(TIF)

Table S1 Excavation time of a tuco-tuco observed outside the enclosure.
(DOC)
