# Supplementary figures and images for: Field and Laboratory Studies Provide Insights into the Meaning of Day-Time Activity in a Subterranean Rodent (Ctenomys aff. knighti), the Tuco-Tuco
Source: PLoS One. 2012 May 23;7(5):e37918. doi: 10.1371/journal.pone.0037918 (PMC3359304; doi:10.1371/journal.pone.0037918)

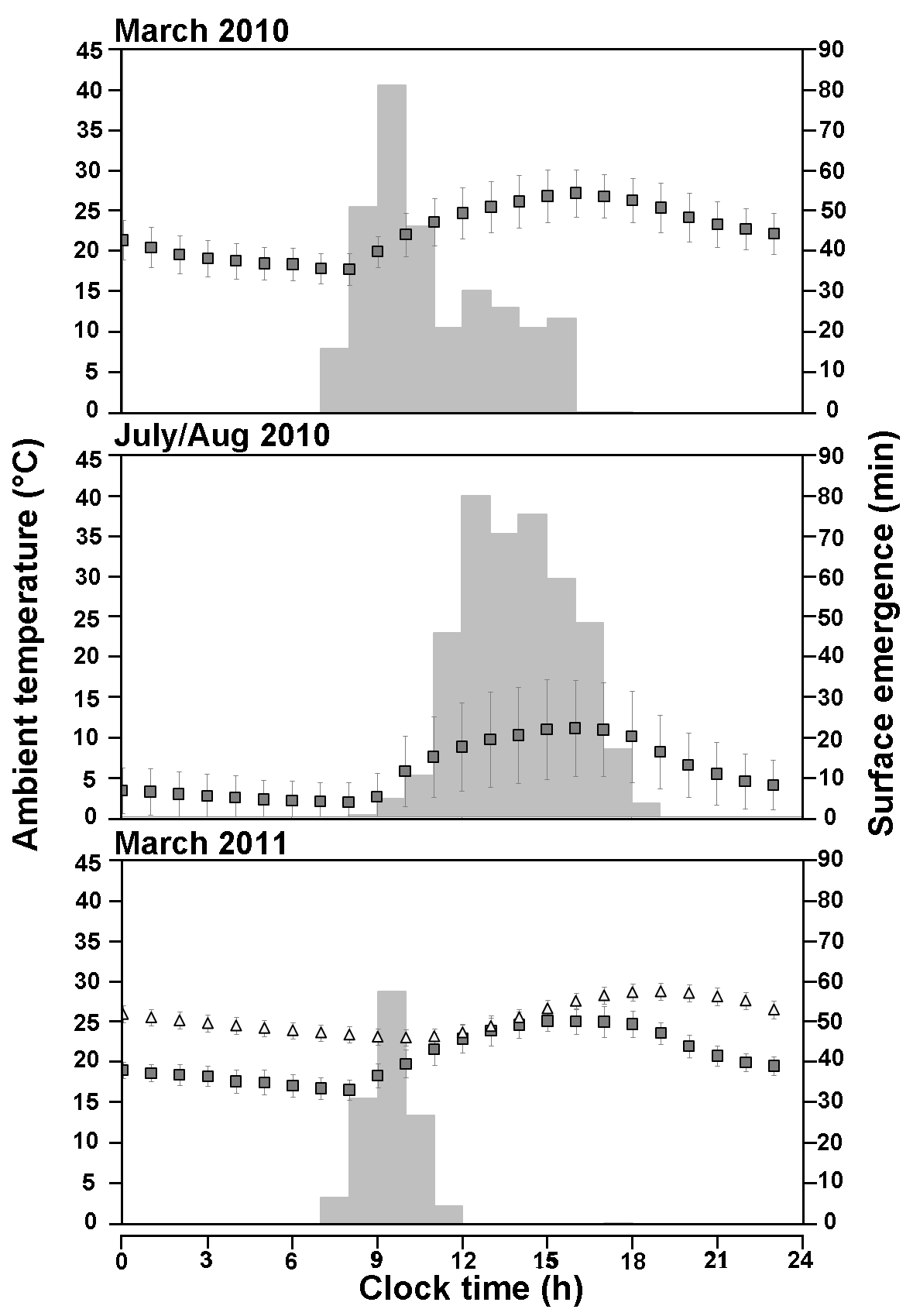

Supplement: Figure S1 — Daily variation of environmental temperatures. Mean values (denoted by squares) of registrations during the days indicated in Table 1: March 2010 (top), July 2010 (middle) and March 2011 (bottom). The underground temperature (60 cm deep) is included in March 2011 data (bottom) (small triangles). The timing and frequency of surface emergences of the tuco-tucos is indicated by vertical bars. (TIF) [file pone.0037918.s001.tif]

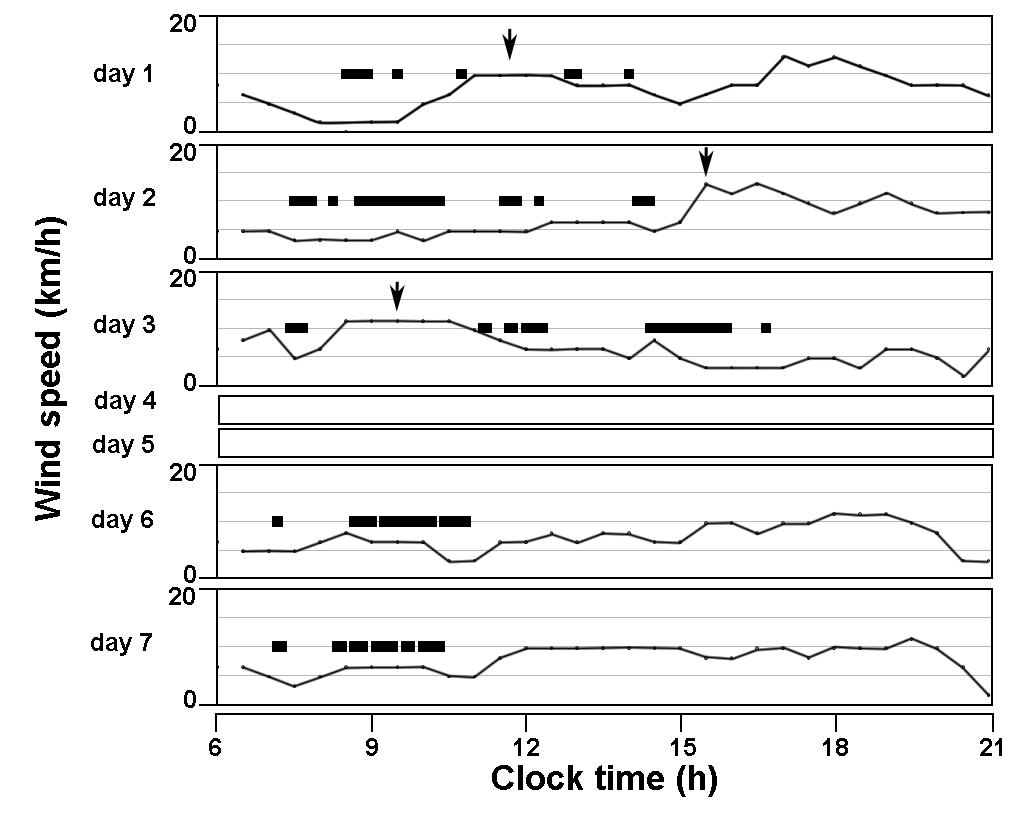

Supplement: Figure S2 — Surface emergence timing (black bars) and wind speed (black line) data for the March 2010 observation. The arrows indicate the moments where wind potentially acted as a masking agent for surface activity. Note: Data on days 4 and 5 was lost, due to equipment failure. (TIF) [file pone.0037918.s002.tif]

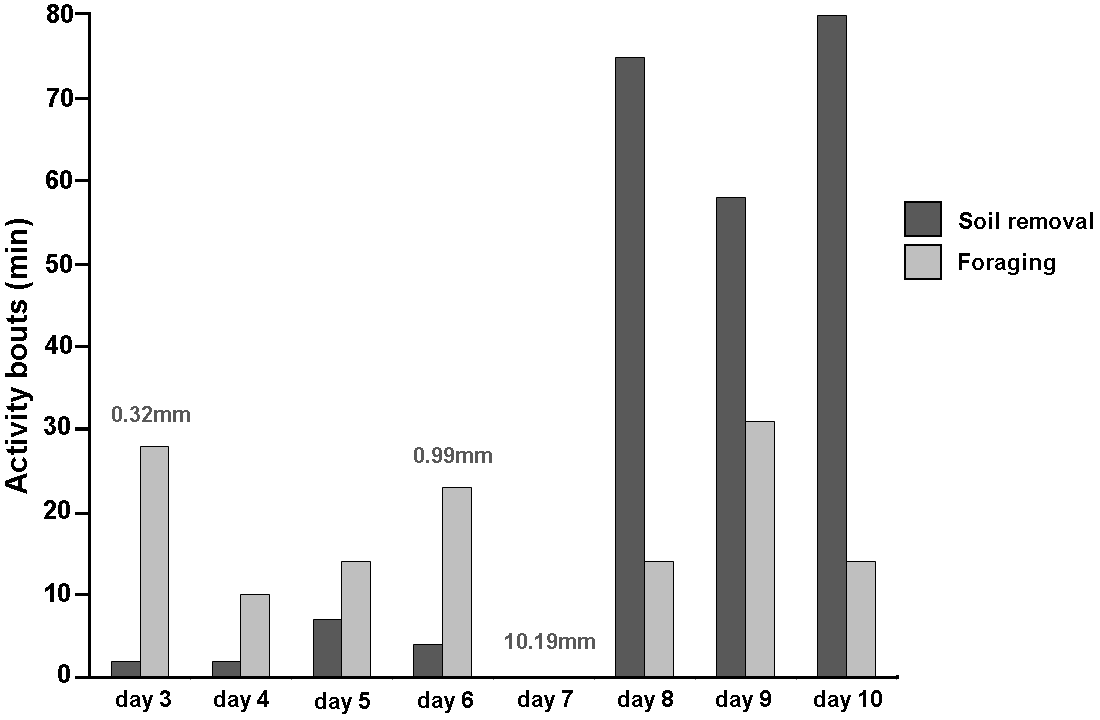

Supplement: Figure S3 — Sum of the total time spent in distinct above-ground activities (soil removal and foraging) during the March 2011 observation. The amount of rain is shown on top of the corresponding bars; no value indicates that no rain was registered. (TIF) [file pone.0037918.s003.tif]
