# Supplementary material for: Field and Laboratory Studies Provide Insights into the Meaning of Day-Time Activity in a Subterranean Rodent (Ctenomys aff. knighti), the Tuco-Tuco
Source: PLoS One. 2012 May 23;7(5):e37918. doi: 10.1371/journal.pone.0037918 (PMC3359304; doi:10.1371/journal.pone.0037918)
Supplement: Table S1 — Excavation time of a tuco-tuco observed outside the enclosure. (DOC) [file pone.0037918.s004.doc]

| 28-07-2010 | | 29-07-10 | | 30-07-10 | |
| --- | --- | --- | --- | --- | --- |
| beginning | end | beginning | end | beginning | end |
| 9:00 | 9:40 | 12:21 | 12:24 | 10:55 | 11:38 |
| 11:57 | 11:58 | 13:52 | 14:17 |  |  |
| 12:18 | 12:44 |  |  |  |  |
| 12:45 | 12:55 |  |  |  |  |
| total: 77 min | | total: 28 min | | total: 43 min | |
